# “You can't tee them up for retirement because we have to go out and win gold medals…”: a Foucauldian exploration of Olympic coaching logic and athletic retirement

**DOI:** 10.3389/fspor.2025.1678053

**Published:** 2025-11-20

**Authors:** Neil Boardman, Luke Jones, John Toner, Zoë Avner

**Affiliations:** 1 Department of Sport, Health and Exercise Science, Faculty of Health Sciences, University of Hull, Hull, United Kingdom; 2 Department for Health, University of Bath, Bath, United Kingdom; 3 Centre for Sport Research, School of Exercise and Nutrition Sciences, Deakin University, Geelong, VIC, Australia

**Keywords:** athletic retirement, sport coaching, Olympics, high-performance sport, Foucault

## Abstract

**Introduction:**

Despite decades of research into the phenomena of athletic retirement, many modern athletes continue to report difficulties in adjusting to life beyond sport. As a result, the issue of athletic retirement has become a significant concern for those working within high-performance settings. However, there remains a distinct lack of critical attention regarding how key actors within athletes' lives (e.g., coaches) shape and impact upon their capacities to adjust to post-sport life.

**Methods:**

In this paper, we address this gap in research by presenting qualitative data from interviews with eight coaches working in the context of Olympic sport in the United Kingdom to examine their perspectives regarding the athlete retirement process.

**Results and Discussion:**

Drawing upon Foucault's conceptualization of disciplinary power-knowledge, our analysis illustrates how Olympic coaches' perspectives and experiences of athletic retirement can be linked to the modernist logic and normalizing pressures that characterize Olympic level coaching environments.

## 1 Introduction

Historically, sport coaches have predominantly been positioned as mediators and facilitators of athlete performance. Indeed, when examining discourses of what it means to be an “effective” coach, we are met with one ever-present truth that involves coaches working to “continually develop innovative and progressive training methods and coaching practices” that contribute to the optimization of athlete development and performance (1, p. 297). To do so, modern sport coaches are expected to draw upon a range of normalized technical, tactical, scientific, and inter- and intra-personal skills and methods. Such approaches include – but are not limited to – periodized frameworks for structuring athlete training and the employment of “athlete-centered” coaching practices (1–4). Typically, those coaches working in the highest performing settings (i.e., Olympic and international sport coaches)^1^ are believed to be incredibly adept at applying and mobilizing such practices and frameworks to drive, improve, and optimize the performance capacities of the athletes they work with (2, 5, 6).

The importance of athlete development and performance to the modern Olympic coaching role cannot be understated (7). However, over the last two decades, a growing body of research has shed significant light on how sport coaches' practices might pervade the lives of athletes beyond their psychological characteristics, their physical attributes, and their technical and tactical proficiencies. Indeed, critical sport coaching scholars have begun to expose how coaches' engagement with normalized, disciplinary practices (see above for examples) might be connected with a series of damaging implications for athletes' health and well-being both during and after their sporting careers. For example, McMahon and colleagues (8) considered how coaches might encourage sportspeople's over-conformity to problematic eating habits throughout their careers, which may result in disordered dietary relations throughout one's life after sport. Moreover, Harvey et al. (9) problematized how coaches' perpetuation of disciplinary athlete norms (i.e., that a sportsperson's sole purpose should be to develop and improve their performances) is linked to the production of negative stigma and the subsequent development of depressive symptoms for sportspeople.

The growing depth of scholarship concerning the effects of normative coaching practices for athletes' lives is becoming increasingly pertinent, given that contemporary research also continues to emphasize how high-performance athletes often experience great difficulties and challenges in adjusting to life after sport (10–12); a consistent finding of athletic retirement research throughout several decades of interdisciplinary research (13–15). Despite this, there remains a significant dearth of research around how key actors (i.e., coaches) in the lives of high-performance sportspeople can work to shape and influence their retirement adjustments and ongoing post-sport experiences (10, 16, 17). Therefore, the purpose of this paper is to further examine the role and position of the high-performance sport coach within the formation of the athlete retirement process. To do so, we draw upon Foucauldian theorization to consider how Olympic coaches' perspectives regarding athletes' retirement processes are influenced by disciplinary power-knowledge relations (18, 19). Indeed, as Foucauldian scholars we recognize power and knowledge to be inextricably linked and, in turn, we understand that the ways in which coaches think, practice, and work with their athletes cannot be separated from their socio-cultural contexts nor relations of power.

In attending to this research agenda, we answer recent calls for further empirical research into the connection between sport coaching and athletic retirement (10, 16, 20). In doing so, we consider how the actions, beliefs, and practices of Olympic-level sport coaches might influence critical elements of the athlete retirement process e.g., preparation for the retirement moment (21).

## 2 The relationship between sport coaching and athletic retirement

Extant research on the phenomena of athletic retirement is relatively extensive and longstanding, particularly within the disciplines of sport psychology (15, 22, 23) and, to a slightly lesser extent, sport sociology (13, 14, 24). Historically, athletic retirement research has predominantly highlighted the often-harsh challenges experienced by sportspeople throughout their lives after sport [see systematic reviews by Fuller (25), Park et al. (26)]. From a psychological standpoint, these difficulties have primarily been tied to individual characteristics, such as the concentration of one's athletic identity (27–29), and the circumstances of an athlete's retirement i.e., whether the retirement “moment” in question was voluntary or involuntary (30, 31). To help mitigate these possible challenges surrounding athletic retirement, researchers have consistently pointed to the importance of sportspeople planning for their adjustments to post-sport life, as well as the significance of mobilizing the transferable competencies developed during a sporting career e.g., emotional resilience and communication skills (32, 33). This is because anticipating and preparing for the retirement transition can allow sportspeople additional time to diversify their educational and occupational experiences, thus offsetting potential associated financial and identity-related challenges (23, 34).

Over the last two decades, a growing body of socio-cultural and psychosocial research has begun to shed light on how high-performance athletes' retirement experiences might be best framed as relational implications of their immersions within, and associated norms of, sport cultures (12, 35, 36). More recently, research emerging from this body of literature has provided insight into how athletes' relationships with key actors within these environments, such as coaches and managers, can influence their transitions into post-sport life (37). Two primary perspectives have driven this insight.

Firstly, research conducted through a psychosocial lens has primarily suggested that exposure to autocratic coaching styles during a sporting career can negatively impact upon athletes' capacities to adjust to retired life (38–40). These scholars have argued that such approaches to coaching, which are often framed as indicative of a lack of “care” in the coach-athlete relationship, serve as precursors for negative retirement experiences as coaches are likely to provide low levels of both informational and emotional support to their transitioning athletes (39). Lavallee and Robinson's (40) research with former female gymnasts suggested that these experiences can lead to issues with premature identity foreclosure and feelings of limited control for athletes around the post-sport adjustment; two key indicators of “problematic” retirement transitions (25, 26).

Secondly, research conducted through a socio-cultural, and predominantly poststructuralist, lens has considered how an exposure to normalizing arrangements of power during a sporting career can impact upon athletes' retirement adjustments (10, 21, 41). Indeed, post-structural scholars have drawn upon Foucauldian theory to consider how the disciplinary logic that sits at the heart of modern coaching methods can problematically shape athletes' retirement experiences – influencing their lived experiences [e.g., (10)], their bodily relations [e.g., (8)], and their movement practices [e.g., (11)] throughout their lives after sport. This body of work represents an important advancement for the field of athletic retirement because it has brought attention to the potentially residual dangers of disciplinary coaching practices for the lives of sportspeople. In doing so, this area of research has led an increasing number of scholars to question how the modernist, disciplinary logic that informs dominant approaches to sport coaching can be further disrupted to more effectively support athletes’ post-sport adjustments (10, 16, 42).

Despite the progress made in recent years to better comprehend the relationship between sport coaching and athletic retirement, scholarly understandings of this connection remain limited (17). One reason for this paucity in research was described by Boardman and colleagues (16, p. 244):

…scholars have attached significant weight to data collected from retired athletes as a method of gaining insight into personal experiences of post-athletic life. As a result, efforts to map the role of the coach in the formation of retirement experiences are limited in terms of the depth of contextualization they offer to the inherently social and relational nature of the high-performance sport landscape.

For the most part, previous research has come at the expense of seeking critical dialogue with coaching practitioners regarding how their biographies, knowledge, and practices influence their perspectives about athletic retirement.

This limitation in extant research has partly been addressed in a recent paper by Brown (20). In this study, Brown, drawing upon the findings of a Foucauldian Discourse Analysis of eight interviews conducted with coaches from the United Kingdom, demonstrated that coaches draw upon dominant performance discourses to construct their perspectives of the athlete retirement process. Most coaches interviewed for Brown's study positioned athletic retirement as inevitably problematic, owing to sportspeople's characteristic adoption of high-performance sport's dominant discourses around performance e.g., the need for self-discipline and self-sacrifice. Alternatively, the remaining coach participants in the study framed the athlete retirement process in more positive terms. These coaches largely perceived the post-sport transition as an opportunity for athletes to redeploy the valuable life skills that are taught through a career in sport (32, 33). Brown (20) argued that his coach participants' stances were typical of patriarchal constructions of coach-athlete relationships, by which the coaches felt responsibility to support their athletes throughout their post-sport adjustments. However, drawing upon Foucault's (68) concept of governmentality, Brown (20) theorized that the responsibility for managing the retirement transition is often placed upon individual athletes. This is because coaches, despite typically feeling a sense of responsibility to support their athletes during this adjustment, are subject to disciplinary tensions to comply with performance discourses and standards, which limits the time and resources they can devote to such duties.

Brown's study covered important new ground in exposing the relational tensions associated with athletes' retirement adjustments. However, as the author clarified in his paper, this study only further re-emphasized the necessity of continuing to investigate the connection between sport coaching and athletic retirement through a post-structural lens. Therefore, in this paper, we build upon the important foundations laid by previous scholarly work to consider how Olympic coaches' perspectives and experiences of athletes' retirement adjustments might be influenced by disciplinary power-knowledge relations (18, 19).

## 3 Foucault, disciplinary logic, and the modern Olympic coach

Michel Foucault (18, 19, 43) was interested in how individuals are shaped by the cultures in which they are immersed. Throughout his scholarly career, Foucault consistently concerned himself with how human beings – their thoughts, their actions, and their bodies – are formed through relations of power. In his earlier work, Foucault (18, 19) set out to trace the emergence of modern power and to explore *how* power functioned in the modern epoch. Foucault (18, p. 94), in a position contrasting the dominant perspectives characterizing structuralist thought at the time, illustrated that power is not a possession to be “held” by a nation-state or economic class, nor is it something that could be “acquired, seized or shared” by these groups. Rather, he considered power to be a relational force that manifests through multiple forms (44). In doing so, Foucault demonstrated how power, through its inseparability to knowledge, pervades all aspects of our modern society. Hence, our lives and experiences are shaped by power-knowledge relations, “where power is subtly played out to influence, objectify, and produce people's thoughts, feelings and actions” (45, p. 125).

Foucault's (18, 19) analyses of sexuality and modern penitentiary systems allowed him to reveal how individual subjects are produced, controlled and normalized via a series of subtle technologies that are diffuse throughout Western societies and cultures. Foucault (19, p. 137) detailed four disciplinary “techniques” (*the art of distributions*; *the control of activity*; *the organization of geneses*; *the composition of forces*) that “made possible the meticulous control of the operations of the body” [see (4) for a detailed explanation of these concepts, mapped to the context of high-performance sport]. For the theorist, these techniques did not operate in isolation, but in combination with three disciplinary instruments (*hierarchical observation*; *normalizing judgement*; *the examination*) and *the confession* to control subjects [(18, 19); see (1) for a detailed analysis of these concepts in the context of high-performance sport coaching]. Foucault (19, p. 136) described in detail how the combination of these technologies worked to render individual bodies “docile”; that is, bodies that are both politically obedient and economically efficient – subjects that “may be subjected, used, transformed and improved”.

Over the last two decades, poststructuralist coaching scholars have consistently shown how Foucault's disciplinary framework can be effectively mapped to the dominant logic and methodologies of modern sport coaches (1, 5, 46). Examples of such disciplinary coaching practices include the meticulous control of athlete activity through highly choreographed training regimen, as well as the perpetual examination of their physiological and psychological attributes via surveillance technologies (45, 47, 48). Historically justified using performance-based and bio-scientific rationale, disciplinary practices have athlete control and the consequential production of their docility at their core (44, 46, 49). Such practices also form the dominant regimes of truth that define modern “effective coaching”, particularly for those operating in high-performance, Olympic-level environments (1, 50).

In recent years, Foucauldian retirement scholars have highlighted how the associated docility-inducing effects of disciplinary coaching practices can have complicating implications for athletes' adjustments to post-sport life (11, 41, 42). In doing so, many of the same thinkers have argued that in order to better support athletes' post-sport adjustments, more must be done to disrupt the disciplinary logic that sits at the heart of knowledge and practice throughout high-performance sport coaching cultures (10, 16). Of course, to do so would be no simple feat. The dominance of disciplinary logic throughout high-performance coaching contexts has led a number of Foucauldian coaching scholars to argue that the widespread over-reliance on such wisdom has also rendered many coaches docile themselves. Indeed, these researchers have convincingly shown that most high-performance coaches, although incredibly adept at mobilizing normative practices, do not tend to see and think beyond the modernist logic that permeates these settings (51–53).

Despite the positive advancements that have been made in the fields of sport coaching and athletic retirement to demonstrate the unfortunate, residual implications of an exposure to disciplinary coaching practices for athletes' retirement experiences, understandings of the interconnections between these phenomena remain limited (16, 17). More specifically, extant research has neglected to consider how disciplinary coaching logic impacts upon practitioners' experiences and perspectives of their athletes' retirement processes and how, as a result, these might obscure associated viewpoints regarding the re-imagination of disciplinary coaching practices for better supporting sportspeople's post-athletic adjustments. For example, re-thinking dominant approaches to orchestrating training and development might allow athletes the additional time and autonomy required to think more effectively about retirement planning (5, 10, 21). We address this paucity in research in this paper.

## 4 Methods

This study was informed by the paradigm of poststructuralism. As post-structural researchers, we understand “knowledge” to be contextual, and “reality” and “truth” to be multiple and subjective (54). What is more, we do not believe that empirical research can be used to reveal a singular truth or reality about our social world (or, in this case, about the relationship between sport coaching and athletic retirement), nor do we deem that researchers can remain neutral or objective throughout the research process (54). Rather, we envision social research to be a vehicle for examining the subjective nature of the social world, as well as a means for considering how the production of knowledge is tied to socio-cultural arrangements and relations of power (55). Therefore, in this study, we aimed to examine how disciplinary power-knowledge relations influence the ways in which high-performance sport coaches understand and experience the retirement processes of the athletes they (have) work(ed) with.

For this study, we conducted semi-structured interviews with eight coaching practitioners. Participants were recruited via a purposeful sampling approach (56). In doing so, our intention was to identify participants that possessed the appropriate characteristics, experiences, and knowledge to partake in a detailed discussion about retirement from high-performance sport from a coaching perspective (57, 58). To recruit participants for this study, we worked closely with a non-academic partner of the first author's host institution at the time of study. The organization acted as a sample “gatekeeper” and provided the first author with the contact details of potential participants, sourced from their networks with national governing bodies in the United Kingdom. Three primary points of criteria were used to determine participant eligibility. These were:

A) Participants must have experience of coaching Olympic athletes in the United Kingdom
B) Participants must have experience of working with British Olympic-level athletes that have announced their intentions to retire from sport during their time as a coach
C) Participants must have experience of working with athletes that have been de-selected from high-performance programming [see UK Sport (59) for a description of current funding structures] during their time as a coach

Coaching practitioners that met criteria point “A”, as well as one (or both) of criteria points “B” or “C” were deemed eligible to participate. The employment of these criteria resulted in the recruitment of eight coaching practitioners. At the time of interviewing, the coach participants were all working in the context of Olympic sport in the United Kingdom, with their experience providing coverage across several sporting disciplines (e.g., cycling, rowing, gymnastics), male and female teams, and coaching roles (e.g., head Olympic coach, coach developer). All participants identified as male and have been provided with a pseudonym. It is important to note that all of this study's participants have significant experience (mean value of 21 years) of working within the context of high-performance and Olympic sport in the United Kingdom – a setting with strong links to high-pressure deliverables (60) and hierarchical structures [see Feddersen et al. (61) for a detailed exploration of ways of working within these cultures].

The first author's University ethics board provided ethical approval for this study (REF: FHS445). The first author conducted one semi-structured interview with each of the eight participants, with an average duration of 75 min; the semi-structured interview guide included several pre-determined and open-ended questions around the topic of study (see below). The interview guide was designed to allow flexibility for additional points of interest arising throughout the conversation to be explored through active listening and probing i.e., in listening carefully and actively to participant responses, the first author often requested clarification or elaboration around certain points in an attempt to seek clarity in the data (58, 62). Mobilizing such techniques allowed interviewees the necessary time and space to discuss the deep, multiple, and complex meanings they ascribe to the topic of athletic retirement (55, 62). All interviews were conducted via Microsoft Teams. Following each interview, the first author produced a “cleaned” version of each interview transcript (55, p. 96). This allowed for the key points of each discussion to be included in each transcript, whilst removing certain utterances that did not provide any form of conceptual interest to the final dataset (e.g., “erm…”). This assisted in creating a more efficient analysis process.

Consistent with our post-structural research approach, our semi-structured interview guide was “Foucauldian inspired” (10, 54, 55). Indeed, our interview guide was devised in accordance with an extensive reading and engagement with the theoretical work of Michel Foucault (18, 19), and most specifically his conceptualization of anatomo-political power relations. In doing so, our aim was to expose how the workings and effects of disciplinary power-knowledge relations might impact upon Olympic coaches' experiences and perspectives of athletic retirement.

Our interview guide directly influenced our approach to data analysis. As post-structural researchers, our aim was not to uncover an objective “truth” within the interview data about the connection between sport coaching and athletic retirement (54). Rather we analyzed our empirical material in accordance with the Foucauldian theoretical framework (55). To do so, we closely followed Markula and Silk's (55) recommendations for post-structural interview data analysis. This involved the identification of initial themes via the Foucauldian-themed interview guide, the analysis of these themes for relevant intersections (i.e., connections between discourses) and discrepancies (i.e., contradictory discourses), and the connection of these themes to relations of power and their effects (19). This approach allowed us to analyze, consider, and present our reading of the interview data via our Foucauldian theoretical framework. The discussion presented in the following section of this paper is a direct result of this methodological process.

## 5 Findings and discussion

When posed with questions regarding athletes' retirement adjustments, a number of coaches in our sample shared perspectives and experiences steeped in disciplinary reasoning (52). As discussed in our methods section, in poststructuralist research, the empirical material and analysis cannot be separated from the theory (55). Therefore, throughout our discussion we connect the identified themes of “Retirement as a Potential Performance Risk” and “The Potential Diversion of Retirement” to Foucault's (19) theoretical concepts of the *techniques of discipline* and *instruments of discipline* (1).

### 5.1 Retirement as a potential performance risk

Athletic retirement researchers have long emphasized the importance of athletes' efforts to carefully plan and prepare for their retirement adjustments (21, 31, 34). When asked to share their perspectives and experiences regarding retirement planning efforts, several of this study's participants suggested that athletes' attempts to prepare for their lives after sport, particularly during the build-up phase to an upcoming competition [see *the examination*; (4, 19)], could result in an increased vulnerability to performance decrements; an issue widely perceived as unaffordable during this period. For instance, Tom remarked that:

“It is really hard. We are working in really distinct cycles, so the peak performance target is about 2 minutes before they usually retire. So, at the point where you would want to tee them up for retirement, you can't tee them up for retirement because we have to go out and win some gold medals as we have to make sure the whole programme stays afloat”

These words represent a cautionary perspective regarding how the disciplinary organization of the coaching process may be disrupted by sportspeople's efforts to prepare for their retirement adjustments. When asked about how training regimen [e.g., *time-tables*; (4, 19)] might be re-organized to allow athletes sufficient time and space to plan for their retirement adjustments, David shared a similar sentiment:

“There is no reason for a person to change anything before you retire if you are successful. People retire, say, after the Olympics but up until the Olympics you will not change anything because you need the consistency.”

These responses are grounded in a disciplinary logic of progression (44). Modern Olympic coaches seek to organize and capitalize upon time in an economical manner with the aim of inducing a state of continuous improvement within their athletes; “a linearity where all progress is oriented to a fixed, stable point” (45, p. 130), such as a competition. In turn, coaches most typically draw upon scientifically informed disciplinary techniques (e.g., periodization) to create and reinforce order, consistency, and predictability in the athlete development and performance processes (5, 63). From the interview extracts above, it is evident that both of these practitioners feel that athletes’ retirement planning efforts might disrupt the sense of consistency cultivated through the application of these accepted, normalized and “effective” progressive techniques. These words could be interpreted as emblematic of these coaches' docility to disciplinary, modernist wisdom (52). This is because the shared perspectives are grounded in a desire for “control” and an associated ambiguity around those phenomena (i.e., athletes' post-sport adjustments) that fall outside of this remit (20, 53). This point is particularly pertinent when considered as an implication of the discursive conditions within which these coaches operate (60, 61). Indeed, as Tom's remarks suggest, Olympic coaches face a series of normalizing pressures to deliver results – and to work in expected, preferred, and normalized (i.e., disciplinary) ways - in line with funding and medal targets (64).

In a slight departure from this line of thinking, when posed with similar questioning, several other coach participants were not totally dismissive of the possibilities regarding the re-choreography of their athlete training and development approaches. Indeed, these coaches, in a manner contrary to David above, indicated that re-thinking dominant planning techniques, such as the timetabling of training sessions (4, 5), *could* have a positive impact upon athletes' capacities to prepare for their retirement adjustments (21). However, these coaches took time to carefully caveat this position by suggesting that their openness to re-thinking their own practices, with respect to these impacts, would be contingent on a series of performance-based variables. Indeed, several participants made it clear that any disruption to their current planning approaches would only be considered under the condition that the individual in question continues to comply with expected performance standards. For example, Roy commented that:

“It was never discouraged to do stuff outside of sport. It was just a challenge to make sure it didn't have such a negative impact…we really had conversations with them about that to ask if it was the right time to do it…only if it doesn't have such a negative impact on the training schedule. What I mean by that is, you know, what is the moment? Is it the week before the Olympic qualifier? Is it in the final run? Is it in the coming weeks before selection? So, I wouldn't always say to them they can go and do whatever they need to do.”

Furthermore, Patrick added that permitting athletes to explore their options beyond sport should be a calculated decision informed by a performance-based equation:

“I think if they have a true desire to explore something else, well I would have the conversation with them about what the cost is of doing that, in relation to their performance.”

Brandon shared a similar thought:

“It is a challenge. If they are training 30 hours a week with a major competition coming up and then doing a course on how to become a barista which means they are standing on their feet for many hours…you absolutely want to encourage stuff like that but you need to encourage it at the right time so it has a minimal impact on performance…In an ideal world, they would say they are thinking about doing this course, but then we can ask if it can be done immediately after the season has ended…and it is not going to be as critical. It does have a big impact, stuff like when they are training and they are constantly going out meeting friends at cafes or going for long walks with their friends, you know, this stuff actually has an impact.”

In the extracts above, these coaches appear hesitant about relinquishing the grasp of those training techniques that combine to produce “a continual growth of control over the body” (44, p. 78). To help arrest their concerns regarding the potential loosening of disciplinary controls, these participants suggested that they would look to question the timing of their athletes' retirement planning efforts. From a Foucauldian perspective, this can be treated as potentially problematic. This is because such approaches, although not necessarily intentional, have the potential to further bind sportspeople to their disciplinary subjectivities, to compound their states of docility, and to reinforce hierarchical coach-athlete relationships (1, 65, 66). Again, this position can be understood as symptomatic of the coach participants' docility. Indeed, most of the Olympic-level coaches interviewed for this study did not evidence attempts to problematize how the intricate workings of power in coach-athlete relationships can work to limit the subjectivities of the athletes they coach (43, 44). This problematic position was particularly evident in Brandon's statement as his words suggest that he perceives the leisure activities of working athletes to be a potential threat to the economic performance and output of their bodies. Although limiting, we do not see this neglect as conscious nor active, but as symptomatic of the prescribed disciplinary logic of high-performance sport coaching (52).

The evidence presented above illustrates that Olympic coaches' compliance with the disciplinary logic of modern sport pervaded their thoughts when challenged about the possible disruption of disciplinary organization (52). Certainly, the evidenced attempts to locate rationality and predictability in the coaching process have perhaps distorted these coaches' understandings of athletes' retirement adjustments. This is because these participants' words primarily point to a stance whereby the prospect of athletic retirement is positioned as a potential threat to the dominant, disciplinary way of doing things in Olympic and high-performance sport, as opposed to a unique opportunity to learn or change their practices (46, 63, 67). We expand on these ideas in the following section of this paper.

### 5.2 The potential diversion of retirement

Foucault (18, 19) theorized that relations of power operate to produce certain types of being congruent with the function of the modern, disciplinary society. Olympic-level sport is a classic example of such a normalizing project [see the *modern disciplines*; (19)] that produces subjects whose bodily and mental subjectivities are intensely fabricated in accordance with their designated *function* i.e., sporting performance (4, 44, 48); a mode of being that can carry complex and contradictory legacies for athletes' ongoing adjustments to post-sport life (10, 11). As previous Foucauldian-informed research has shown, sport coaches can be complicit in shaping such problematic subjectivities, owing to their normalized engagement with those practices that resemble the *instruments of discipline* (1, 4, 18, 19). This is because the cultivation of disciplinary modes of being is widely accepted as an efficient method for preparing sportspeople for the rigors and demands of Olympic-level competition. With this in mind, how do these discursive understandings of the modern sportsperson impact upon coaches' perspectives of the athlete retirement process?

Considering the data presented in the previous section of this paper, it is evident that this study's participants are cognizant, and often cautious, of how athletes' considerations of post-sport life might interrupt the progressive logic that is tied to the pursuit of high-performance (44). Such reasoning is very much reflective of these coaches' fine attunement with discourses of modern effective coaching that emphasize the importance of athlete preservation i.e., limiting potential distractions to optimize the longevity and efficiency of athlete performance (1, 21, 60). Given the significance attached to the cultivation of disciplinary subjectivities in modern sport, several coach participants displayed a sense of hesitancy when asked about how athletes' attentiveness to their post-sport futures may disrupt those highly calculated mentalities that they and their organizations have long focused on inscribing (21, 37). For instance, Tom conceded that:

“In terms of discussing life beyond sport, they tend not to go there with it and I wouldn’t push it either because they are focused on what they’re doing…I think there is relevance in making sure they are teed up for retirement…My hesitance around it is that if we show all the goodies you can have in retirement, does it draw their focus?”

In addition, Simon noted that:

“The danger is that they would make a decision like that and then think now they can just relax because the decision has been made…It is not an avenue to start cutting corners…you would want to make sure that anyone, whether they were retiring or not, made the most of whatever opportunity they have.”

In these interview extracts, the coaches express concerns regarding the ways in which the prospects and challenges of retirement may work to distract athletes' foci during the latter stages of an Olympic training cycle; a position which might be understood as a contestation to those discourses which emphasize the criticality of retirement planning and consideration (21, 34). These concerns echo Denison's (46) warning that when faced with possible issues relating to athlete performance and development, coaches typically attempt to locate the “problem” within the psychological characteristics of the individual in question. Denison argued that this characteristic position often comes at the expense of coaches actively problematizing how their own practices may remain genuinely “effective” in the respective context. This represents another element of the coach participants’ perspectives that might be considered as emblematic of their docility. Indeed, Tom and Simon appeared to find difficulty in thinking beyond the disciplinary wisdom that athletes can only perform at their optimal level should they remain docile, determined, and focused upon a singular objective (4, 9, 52). It is these very modes of thought that place athletes under a permanent gaze to consolidate their disciplinary subjectivities in ways that impede upon their capacities to think actively and effectively about retirement preparation (21).

To help arrest their concerns around the deviation of athletes' mindsets during the build-up to their post-sport adjustments, several of the Olympic coaches suggested that they would look to engage in conversations with their athletes to access a deeper understanding of their modes of being and thought during this period. For instance, Simon mentioned that:

“The main thing I would be talking to the athlete about in that scenario is, you know, that I understand that this decision has been made but you need to understand that you need to be fully in. It can't be that you are biding time until you go off and do the rest of your life. You have to be 100% committed to making the most of this opportunity.”

In a similar vein, Richard noted that:

“I think I’d be having a conversation with them first and foremost…I think having that conversation is the biggest thing for me and making sure that they’re not too long in a place of indecision. I think that is where things start to go wrong – when people are a bit unsure. If they’re going into an intense competition whilst they are feeling unsure about if that's what they even wanna do or not, then that's not good because at the end of the day…you need to be prepared to a good level.”

Furthermore, Brandon shared the following words:

“You would be aware that they were going to retire after that point and you would be a bit more aware of finding out that their motivation was still there…you might have different conversations with them…if they are on programme and they told me they were stopping after the [competition] but they didn't plan to go there and were just actually running out their contract, then that would be a problem. But, if they are saying they are going to the [competition] and they are going to try and win a medal, then there is no change. It makes no difference that they are going to retire afterwards.”

The responses above suggest that each of these coaches rely upon the foundations of coach-athlete relationships to develop an understanding of the subjectivities of the athletes they work with on a rich, detailed, and individualized basis (65). Indeed, these participants indicated that the trusting relationships they hold with individual athletes might act as an avenue for formulating deep, informed readings of their modes of being and thought, with respect to their impending retirement adjustments. The participants' outlined intentions are representative of an approach grounded in care, athlete preservation, and protection. Indeed, as the practice of modern “effective” coaching requires coaches to gather and apply professional and personal knowledge about athletes to support their competence and performance, the mobilization of such approaches has become an accepted method for Olympic-level coaches. Clearly, these coaches are well-acquainted with these “athlete-centered”, “holistic” methods (1, 52).

Parallels can be drawn here between the coaches' outlined approaches and Foucault's (18) concept of *the confession*. In the scenarios described above, *the confessor* (i.e., the athlete) would be required to divulge their *personal history* (i.e., the state of their psychological focus upon an upcoming competition) to *the expert* (i.e., the coach), so that judgement could be passed on the conditions of their mentalities (i.e., maintaining a total commitment to the pursuit and delivery of high-performance) prior to retirement. In doing so, the coaching practitioners would be able to inspect and detect any potential abnormalities in the athletes' subjectivities during the critical period that precedes *the examination* i.e., the upcoming Olympic Games or major competition (18, 19, 44).

As previous Foucauldian coaching research has shown, the mobilization of confessional techniques can have unintended yet often problematic implications for sportspeople, with respect to both their in- and post-sport lives (1, 65, 66). This is because upon an athlete confessing their state of normalcy to their coach, a discursive hierarchy is reinforced, whereby the coach is granted an authority to discipline, punish, and attempt to “fix” the psychological “deficits” of the respective individual (10, 46, 66). Therefore, what might first appear a “rational” or “caring” approach to working with retiring athletes might also have a series of unintended and negative consequences for their lives and forthcoming post-sport adjustments (65). In using a Foucauldian lens to analyze and theorize the responses above, we have presented a problematized reading of those primarily psychosocial understandings of athletic retirement which have suggested that immersion within “positive” and “caring” coach-athlete relationships is more likely to result in 'smoother’ or more “productive” retirement adjustments [e.g., (39)]. Indeed, we, alongside many of our Foucauldian influenced peers, would argue that it is important not to uncritically accept such positions but to emphasize the hidden and potentially negative consequences of such practices, even if they are unintended (1, 65).

## 6 Conclusion

In this paper, we have presented a reading of the connection between sport coaching and athletic retirement informed by a Foucauldian theoretical framework. In doing so, our analysis has illustrated that Olympic coaching practitioners' perspectives and previous experiences around their athletes' retirement processes can be connected to the disciplinary logic that pervades modern high-performance sport (52). That is, throughout their considerations, the coaches interviewed for this study consistently sought to locate how the athletic retirement process might act as a potential threat to the dominant ways of working and thinking in Olympic sport settings. Namely, the coach participants often displayed hesitation with regard to how athletes' deliberations regarding their post-sport futures may disrupt the rationality, consistency, and predictability that they seek to cultivate throughout the performance and development processes (2, 63). In response, several participants described how they might draw upon related disciplinary instruments to help mitigate these concerns (1).

Furthermore, our analysis has pointed to how these disciplinary ways of thinking, informed by dominant discourses of “effective coaching” and the normalizing pressures that Olympic coaches face to coach in these ways, have perhaps constrained these Olympic coaches' capacities to think critically about their roles in the athlete retirement process (1, 52). Indeed, when posed with questions regarding how dominant athlete performance and development techniques might be disrupted in order to better support sportspeople's retirement adjustments, the coach participants' responses were predominantly imbued with disciplinary and performance-based reasoning (53). These findings support the argument made by Denison and colleagues (52, p. 780) that, within the tightly organized and controlled parameters of modern high-performance coaching frameworks, “there is almost no space for a coach to generate alternative views, knowledge or practices”. In turn, coaches become docile to such logic and encounter great difficulty in thinking beyond disciplinary limits when presented with challenging and important questions around the residual impacts of disciplinary practices for athletes' lives (20, 51, 52).

This paper has made progress in answering Boardman and colleagues' (16), Brown's (20) and Jones and Denison's (10) calls for further research into the relationship between sport coaching and athletic retirement. However, we do believe that we have only begun to scratch the surface in exposing and understanding the nuances of these connections and, in turn, we would argue that further research into this relationship remains a necessity to better support athletes' post-sport adjustments in the future. With Foucauldian logic acting as a suitable heuristic device for doing so, we believe that energy into this research agenda would be best spent in two ways. Firstly, following Gearity and colleagues' (65, p. 24) call for scholars to “cease…in describing the effects of caring in uncritical, positive terms”, further research should focus on critiquing and de-constructing the emerging psychosocial discourses that have placed “positive”, “humanist”, and “caring” coaching approaches at the heart of enhancing support for retiring athletes (39). This is because such lines of thought neglect to consider how the associated approaches might also lead to the further subordination of sportspeople, the intensification of their states of docility, and the complication of their long-term adjustments to post-sport life (1, 10, 66).

Secondly, further research should extend the Foucauldian lens of problematization deeper into Olympic sport's hierarchy of leaders. It has become clear in this study that modern sport coaches are subject to a series of normalizing pressures to comply with expected and preferred ways of coaching (64); methods to which they have become docile. Therefore, we believe that it is critical to invite a wider network of subjects who hold ethical responsibilities to the lives of athletes (e.g., coach developers, performance analysts, player care and welfare officers, performance lifestyle practitioners, scouts) into these conversations around athletic retirement. This would allow for a more informed consideration of how coaches' perspectives and experiences of the athlete retirement process are influenced, and perhaps constrained, by the wider discursive elements of the Olympic sport landscape e.g., funding structures and medal targets. Furthermore, given that all participants in this study identified as male, we see it as pertinent to extend this invite towards female Olympic coaches in an effort to better consider the potential impact of gendered dynamics upon coaches' perspectives of athletic retirement.

## Data Availability

The original contributions presented in the study are included in the article/Supplementary Material, further inquiries can be directed to the corresponding author.

## Footnotes

1 In line with Jones and colleagues (11, p. 2), we define high-performance sport as “a fulltime competitive bodily endeavor, typified by the representation of a community group/institution and compensated by finance (including scholarships) and/or status, access, and affiliation”.
